# The private well water climate impact index: Characterization of community-level climate-related hazards and vulnerability in the continental United States^☆^

**DOI:** 10.1016/j.scitotenv.2024.177409

**Published:** 2024-11-14

**Authors:** Komal Peer, Brian Hubbard, Michele Monti, Patrick Vander Kelen, Angela K. Werner

**Affiliations:** a National Environmental Public Health Tracking Program, Division of Environmental Health Science and Practice, National Center for Environmental Health, Centers for Disease Control and Prevention, Atlanta, GA, United States; b Environmental Health Services Program, Division of Environmental Health Science and Practice, National Center for Environmental Health, Centers for Disease Control and Prevention, Atlanta, GA, United States

**Keywords:** Tracking, Drinking water, Water Wells, Spatial analysis, Public health surveillance, Climate change

## Abstract

**Background:**

Private wells use groundwater as their source and their drinking water quality is unregulated in the United States at the federal level. Due to the lack of water quality regulations, those reliant on private wells have the responsibility of ensuring that the water is safe to drink. Where extreme weather is projected to increase with climate change, contamination due to climate-related hazards adds further layers of complexity for those relying on private wells. We sought to characterize community-level climate-related hazards and vulnerability for persons dependent on private wells in the continental United States (CONUS). Additional objectives of this work were to quantify the burden to private well water communities by climate region and demographic group.

**Methods:**

Grounded in the latest climate change framework and private well water literature, we created the Private Well Water Climate Impact Index (PWWCII). We searched the literature and identified nationally consistent, publicly available, sub-county data to build Overall, Drought, Flood, and Wildfire PWWCIIs at the national and state scales. We adapted the technical construction of this relative index from the California Communities Environmental Health Screening Tool (CalEnviroScreen 4.0).

**Results:**

The distribution of climate-related impact census tracts varied across CONUS by nationally-normed PWWCII type. Compared to the Southeast where the majority of the 2010 estimated U.S. private well water population lived, the estimated persons dependent upon private well water living in the West had an increased odds of living in higher impact census tracts for the Overall, Drought, and Wildfire PWWCIIs across CONUS. Compared to non-Hispanic White persons, non-Hispanic American Indian and Alaska Native (AI/AN) persons had an increased odds of living in higher impact census tracts for all four PWWCII types across CONUS.

**Conclusions:**

The PWWCII fills a gap as it provides a baseline understanding of potential climate-related impacts to communities reliant on private well water across CONUS.

## Graphical Abstract

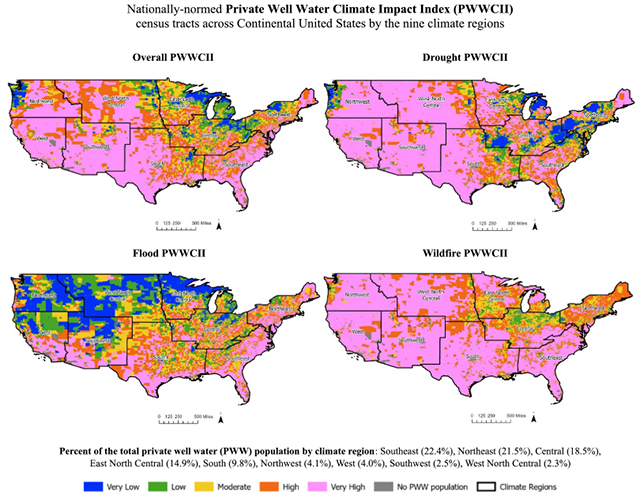

## 1. Introduction

Private wells, also known as domestic or household wells, are groundwater sources that are not federally regulated for drinking water quality in the United States. More than 43 million people, approximately 15 % of the population, rely on private wells as their source of drinking water (USGS, 2019). At the federal level, the Safe Drinking Water Act regulates the quality and safety of drinking water for public water systems only, and in most states, there is limited legislation regulating the quality and safety of drinking water from private wells (USGS, 2019; Bowen et al., 2019). However, private wells are susceptible to physical damage as well as microbiological, chemical, and radiological contamination from a variety of sources (CDC, 2022a) such as septic tanks (Schaider et al., 2016), farm applications and animal waste (Allevi et al., 2013; Manley et al., 2022), climate incidents likeprecipitation (Procopio et al., 2017), and naturally occurring metals and minerals (Arienzo et al., 2022). As a result, household members dependent upon private well water are vulnerable to potential health effects from contaminants, and infants, pregnant women, older adults, and persons who are immunocompromised may be especially susceptible to illness (CDC, 2022).

Due to the lack of water quality regulations for private wells, the responsibility of ensuring that the water is safe to drink falls to those reliant on private wells as their drinking water source (USGS, 2019). This requires a level of ongoing vigilance to test and monitor for contamination and subsequently implement any necessary treatment or disinfection that is often challenging and costly to do (MacDonald and Tippett, 2020). In the case of renters, they may be in a position of undue burden such that they may not be able to take the necessary actions to ensure safe drinking water. Where extreme weather is projected to increase with climate change, contamination due to climate-related hazards adds further layers of complexity to assure the quality of drinking water from private wells (Rose et al., 2001; McMichael et al., 2006; Eccles et al., 2017). Furthermore, new research continues to highlight the need for private well recovery assistance after natural disasters, such as hurricanes (Pieper et al., 2021) and wildfires (Proctor et al., 2020), and new knowledge such as the potentially adverse impact of drought on arsenic exposure from private wells (Lombard et al., 2021a). Screening of communities reliant on private wells and likely to be impacted by well contamination from climate incidents may inform public assistance in the context of preparation for and response to natural disasters as demonstrated by a study conducted in Ireland (Musacchio et al., 2021).

However, significant data challenges exist within the private well water and public health field. Private well water data is highly variable across the nation and is often lacking, incomplete, or inconsistent at best (Backer and Tosta, 2011; MacDonald Gibson and Pieper, 2017). Recently modeled data on the location and contamination of private wells (Johnson et al., 2019; Ransom et al., 2022; Lombard et al., 2021b; Murray et al., 2021), available nationally and at a sub-county scale, presented an opportunity to advance public health practice and research in this area. Hazard data (i.e., climate and contaminant data) alone are insufficient to address the multifaceted issue of climate-related private well water quality. Well maintenance, including well testing and treating well water, are impacted by socioeconomic status and income (Flanagan et al., 2016). While data sources and models (Lombard et al., 2021a; Luh et al., 2015; Tee Lewis et al., 2023) on private well water contamination, natural hazards, and/or social vulnerability exist, to our knowledge, there is no tool that combines all three of these disparate data to characterize communities at the census tract level. Such a tool would provide context about the potential impacts faced by households dependent on private wells within communities to inform conversations, actions, and collaboration among communities and their partners to mitigate impacts.

The U.S. Centers for Disease Control and Prevention’s (CDC) National Environmental Public Health Tracking Program (Tracking Program) and the Environmental Health Services Program (EHS) recognized an opportunity to fill a gap and characterize community-level climate-related hazards and vulnerability for households dependent on private wells in the continental United States (CONUS) by creating the novel Private Well Water Climate Impact Index (PWWCII). Our index was 1) grounded in climate-related priorities of state, tribal, local, and territorial (STLT) public health programs as well as a search of the literature on well water quality, natural hazards, and data modeling; and 2) built with national, sub-county data. The PWWCII was designed as a relative measure to inform both research and practice. Additional objectives of this work were to quantify the burden of climate-related hazards to private well water communities across climate regions and demographic groups as a first step in understanding this often under researched and under addressed area of drinking water.

## 2. Methods

### 2.1. Scope of index

Wildfires and hurricane-related flooding are two events prioritized by STLT public health agencies, and important for CDC’s EHS program. The impact of drought on well water contamination was identified during a literature review and is detailed in Section 2.2. These are also priorities within CDC’s National Center for Environmental Health (NCEH)—a center that provides expertise and leadership in surveillance, epidemiology, technical assistance, training, and preparedness activities related to natural and human-induced disasters to domestic and international partners.

The PWWCII includes national, sub-county private well data for arsenic and nitrate concentrations. To briefly contextualize methodological decisions about contaminants, the potential for human exposure to and health impacts from nitrate and arsenic entering private wells may be exacerbated by the occurrence of flooding and drought respectively, and their presence or magnitude may serve as an indirect marker for the mobilization (or the inverse) of primary contaminants and/or other contaminants of concern for which no national, sub-county data is available. The justification for inclusion of these two contaminants was based on the literature and data search results detailed in Section 3.1.

### 2.2. Literature search and identification of nationally consistent publicly available data

We searched PubMed for articles on well water quality, natural hazards, and data modeling during the fall of 2021. Combinations of the following search terms were used: “private well water,” “domestic well water,” “natural disaster model,” “vulnerability model,” “catastrophe model,” “natural disaster,” “wildfire,” “hurricane,” “flooding,” and “drought”. Papers were identified from the search as relevant to the objectives of this work if they assessed risk or impact of contamination to private drinking water due to a natural hazard; additional papers that met the same criteria or provided nationwide private well water data were identified from the references of the relevant papers. As part of its mission, CDC’s Tracking Program provides nationally consistent, publicly available, sub-county spatially and temporally resolved public health and environmental data that drives data-informed decision making and action to improve community health throughout the country. Guided by this mission, governmental and academic data sources were identified from the literature search as well as from concurrent searches of governmental websites to obtain current, novel, and the highest spatially resolved data for the nation on private wells, natural disasters, and natural disaster relevant private well water contamination. Federal governmental website searches included CDC, Federal Emergency Management Agency (FEMA), Environmental Protection Agency (EPA), United States Geological Survey (USGS), and Drought.gov (a product of the National Oceanic and Atmospheric Administration’s [NOAA] National Integrated Drought Information System [NIDIS]). One author identified relevant papers as well as data and presented the extracted details for review by and discussion among all authors.

### 2.3. Geospatial processing of nationally consistent publicly available data

Prior to index creation, individual spatial variables identified from the FEMA National Risk Index (NRI), CDC Social Vulnerability Index (SVI), and USGS datasets were geospatially processed in ESRI ArcGIS Pro 2.6.7 (Redlands, CA) (Table 1). Briefly, Table 1 variables include *drought:* annualized frequency of drought occurrences (incident-days), *arsenic in private well water:* probability of arsenic concentrations exceeding 10 μg/L in private wells, *coastal flooding:* annualized frequency of coastal flooding incidents, *hurricane:* annualized frequency of hurricane incidents, *riverine flooding:* annualized frequency of riverine flooding occurrences (incident-days), *nitrate in private well water:* groundwater nitrate concentrations supplying private wells (mg/L), *wildfire:* area-weighted burn probability due to a large fire incident, *nationally-normed and state-normed SVI:* sum of 15 vulnerability variables’ percentile rankings, and *private well water population:* 2010 estimated persons dependent upon private well water.

The base spatial layer used for analysis was the CDC Tracking Program’s 2010 census tract shapefile restricted to the continental United States (CONUS). This resulted in 72,392 census tracts. We joined the 2017 FEMA NRI (Zuzak et al., 2021) and 2018 CDC SVI (Centers for Disease Control and Prevention/ Agency for Toxic Substances and Disease Registry/ Geospatial Research A, and Services Program, 2018) census tract datasets to the 2010 shapefile based on census tract FIPS codes. There were 25 census tracts from the 2018 CDC SVI file that required a manual spatial join because they did not join based on FIPS code. The USGS datasets were raster datasets per square kilometer (Ransom et al., 2021; Lombard, 2021; Johnson and Belitz, 2010). These data were converted to 2010 census tract measures (i.e., area-weighted persons dependent on private wells per 2010 census tract, area-weighted average nitrate concentration in mg/L per 2010 census tract, and area-weighted average probability of exposure to arsenic concentrations >10 μg/L per 2010 census tract) using the following geoprocessing tools prior to spatially joining to the CDC Tracking Program’s 2010 census tract shapefile: Raster to Point, Buffer, and Feature Envelope to Polygon. The final processed dataset was exported to SAS 9.4 (Cary, NC) for index creation and analysis, and bar charts were created in Microsoft Excel. Please see the Discussion section for data limitations.

### 2.4. Framework, model approach, and index creation

**Fig. 1.**
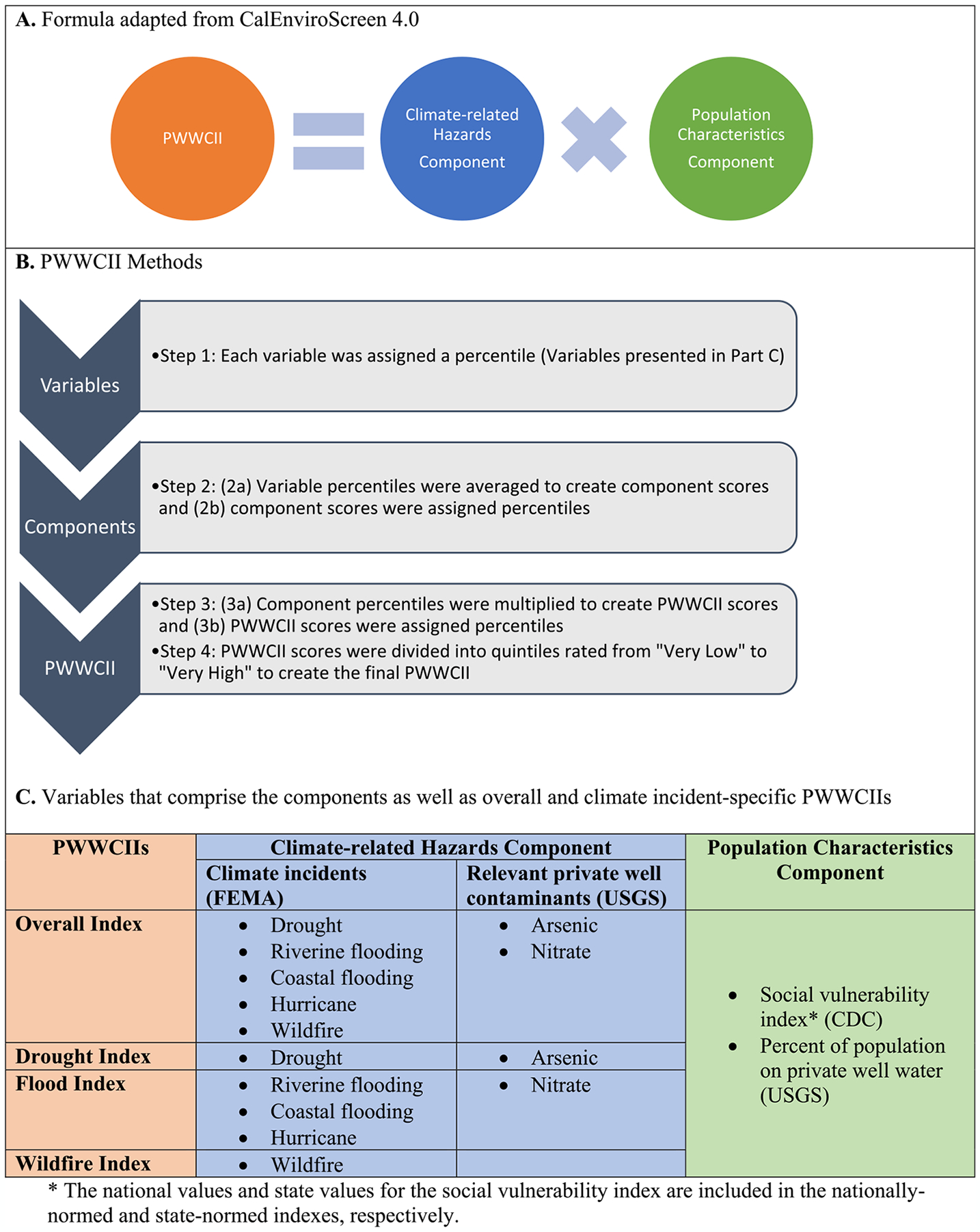
PWWCII formula, methods, and comprising variables.

The latest framework endorsed by the Intergovernmental Panel on Climate Change’s (IPCC) 6th Assessment Report defines risk of climate change impacts as arising from the dynamic interactions of hazards (the potential occurrence of an event or trend that may impact health) with exposure (the presence of people in a place that could be affected) and vulnerability (the propensity to be adversely affected) (IPCC, 2022). While the framework is not prescriptive, risk and impact assessment models in the disaster and environmental health literature (Brody et al., 2012; Blaikie et al., 2014; Zuzak et al., 2022) and grey literatures (CalEPA, 2021; University of Washington Department of Environmental & Occupational Health Sciences and Washington State Department of Health, 2022; Gill et al., 2014) are based upon a common risk equation: risk=hazard×vulnerability. Of these models, CalEnviroScreen’s cumulative impact assessment tool (Version 4.0) characterizes pollution burdens and vulnerabilities affecting California communities at the census tract level with the goal of identifying communities that may benefit from policy change, investment, or intervention. Striving for a similar goal, we adapted the formula and percentile approach underlying CalEnviroScreen 4.0 to characterize climate-related hazards and vulnerabilities for persons dependent on private wells within communities at the census tract level. A summary and the details of our methods are presented in Fig. 1. The formula for this work is:

Climate-relatedhazardscomponent*Populationcharacteristicscomponent=PWWCII

Variables (detailed in Table 1 and Section 2.3) were aggregated into components and then indexes (Fig. 1C). We created an Overall PWWCII and three climate incident-specific indexes—Drought, Flood, and Wildfire. Overall PWWCII included data on drought, flood, and wildfire (Fig. 1).

#### PWWCII Scores (Equation Box 1):

Specific to our purposes, the climate-related hazards component includes climate incidents and relevant private well contaminants, and the population characteristics component includes characteristics at the community level that result in increased vulnerability to these hazards (Fig. 1). First, each variable was assigned a percentile (SAS procedure PROC RANK with the default setting of TIES = mean was used). Second, variable percentiles in the corresponding components were averaged to create component scores; component scores were then assigned percentiles. Third, component percentiles scores corresponding to each index type were multiplied to create PWWCII scores; PWWCII scores were assigned percentiles.

#### PWWCIIs (Equation Box 2):

Fourth, the final impact indexes resulted in five ratings based on quantiles and indicated whether a census tract has “Very Low,” “Low,” “Moderate,” “High,” and “Very High” climate-related impact and are based on the PWWCII scores of a census tract.

#### Geographic coverage:

Census tracts with no population according to the 2010 U.S. Decennial Census or with less than one estimated person on private well water were excluded from index creation. Of the 72,392 CONUS census tracts, the following tracts were excluded to result in a PWWCII dataset of 56,525 census tracts (78.1 %): 350 census tracts had zero population (0.5 %), 8880 census tracts had zero estimated persons dependent on private wells (12.2 %), and 6637 census tracts had between 0.01 and 0.99 estimated persons dependent on private wells per census tract (9.2 %). From here on, these excluded census tracts are referred to as “census tracts with no estimated persons dependent on private wells.” Of these 56,525 census tracts, 235 tracts lacked original source data (231 had missing SVI data and four had missing nitrate data) and as a result, percentile rankings and PWWCIIs are also missing. Thus, 56,290 census tracts were assigned Overall and Flood PWWCIIs, and 56,294 census tracts were assigned Drought and Wildfire PWWCIIs. Lastly, for 146 census tracts, the calculated percent of 2010 estimated U.S. persons dependent on private wells exceeded 100 % of the 2010 census total U.S. population and as a result were top coded to 100 %.

### 2.5. Index evaluation

There is no published measure that currently captures the same construct that our novel index is intended to represent: the potential impact of climate on communities reliant on private well water. We assessed the nationally-normed indexes for construct validity, which is comprised of divergent and convergent validity. Divergent validity assesses to what extent two items measure different constructs, whereas convergent validity demonstrates whether two items measure the same construct. To do this, we examined correlations between our indexes and the 2008–2012 American Community Survey (ACS) variables on owner and renter occupied housing units with telephone service to assess divergent validity as we expected these constructs to be unrelated (Manson et al., 2022). For convergent validity, there is a new index, the climate vulnerability index (CVI), which characterizes community-level vulnerability to and risk from climate change and is intended to inform future research as well as prioritize resources and interventions (Tee Lewis et al., 2023). Therefore, this was considered the most comparable surrogate to our index. Lastly, according to index creation best practices, we assessed that the variables comprising each index were not highly correlated. In regards to construct validity, DeVon et al. (2007) suggest a correlation coefficient of 0.45 or greater to support convergent validity, and their literature review contained some studies that supported convergent validity at 0.30 or greater; below these correlation coefficient values was considered to be in support of divergent validity (DeVon et al., 2007).

### 2.6. Climate regional and demographics analyses

To better understand climate-related impact burden to communities reliant on private well water across CONUS, the nationally-normed Overall and climate incident-specific indexes were examined by climate region and demographics. To examine these differences, conducting chi-square tests followed by post-hoc testing would result in all statistically significant differences due to the large populations being assessed (over 37 million estimated for the U.S. private well water population and over 306 million estimated for the U.S. population). Therefore, Cramer’s V was used to assess univariate associations between PWWCII categories (Very Low, Low, Moderate, High, and Very High) and climate region and demographics. If Cramer’s V indicated an association, simple ordinal regression was performed as post-hoc analysis. Utilizing the Score Test for Proportional Odds Assumption in SAS from PROC LOGISTIC would result in all statistically significant violations to the proportional odds assumption for ordinal regression models again due to the large populations being assessed. Therefore, plots of cumulative logits were generated for each model and examined to ensure cumulative logit curves were relatively parallel to meet the proportional odds assumption.

Climate region data were obtained from NOAA (NOAA, 2004) and census-tract level demographic data were obtained from the 2010 U.S. Decennial Census (Manson et al., 2022). The 2010 estimated U.S. persons dependent on private wells was examined by climate region (Northwest, West, Southwest, West North Central, South, East North Central, Central, Southeast, Northeast). Because demographic information for the 2010 estimated U.S. persons dependent on private wells were not available, we used the 2010 U.S. Decennial Census to examine the total U.S. population by sex (male and female), age (under age 5, 5–24, 25–64, 65 and older), and race/ethnicity (Hispanic, non-Hispanic (NH) Black, NH White, NH Asian, NH Native Hawaiian and Other Pacific Islander, NH Other, NH American Indian and Alaskan Native, and NH Two or more races).

### 2.7. State-normed impact indexes

The methods in Section 2.4 produced nationally-normed climate-related impact indexes that compare census tracts across CONUS and highlight national differences. These methods were repeated to create state-normed impact indexes that compare census tracts within states and are relevant to public health equity work within states; state-normed impact indices can highlight the disproportionate burden of potential climate impacts to communities reliant on private well water within a state.

## 3. Results

### 3.1. Literature search and identification of nationally consistent publicly available data

Four papers were identified from the literature search as relevant to the objectives of this work because they met the following criteria: assessed risk or impact of contamination to private drinking water due to a natural hazard (Pieper et al., 2021; Lombard et al., 2021a; Eisenhauer et al., 2016; Wing et al., 2002); we identified one additional paper providing nationwide private well water location data from the references of the relevant papers (Johnson et al., 2019).

Private well water testing for total coliform and *E. coli* are encouraged to screen and detect, respectively, for potential water-borne gastrointestinal illnesses from microbial contamination due to human or animal waste exacerbated by flooding events (Eccles et al., 2017; Pieper et al., 2021; EPA, 2024a). Testing for nitrate similarly indicates the presence of human and animal waste (from such sources as septic tanks or agriculture) as well as fertilizer contamination in private wells, and nitrates can affect everyone but can cause a serious condition called blue baby syndrome in infants and birth defects in developing fetuses (EPA, 2024b). Studies have demonstrated the impact of flooding or heavy rainfall on animal waste and the contamination of private drinking water (Eisenhauer et al., 2016; Wing et al., 2002; EPA, 2024b) and nitrate was predictive of fecal coliform/*E. coli* presence after precipitation in New Jersey (Procopio et al., 2017). While nationally consistent, sub-county total coliform and *E. coli* data are lacking, nitrate data were available and used as an indicator of potential contamination of private wells due to flooding (Ransom et al., 2022).

Lastly, chronic exposure to arsenic is associated with several cancers, such as lung, bladder, and prostate as well as adverse birth outcomes (Lombard et al., 2021a). Drought can contribute to groundwater wells running dry (Jasechko and Perrone, 2020), and a recent study indicated the potential adverse impact of drought on arsenic exposure via private drinking water wells; because national scale, sub-county data are available, arsenic data were included (Lombard et al., 2021b). Table 1 presents the results of the identification of nationwide, publicly available data sources at sub-county spatial resolution on private wells, private well water contamination relevant to natural hazards, natural disaster occurrence, as well as social vulnerability to natural disasters from governmental and academic data sources identified from the literature search as well as from searches of governmental websites.

### 3.2. Indexes

Fig. 1C summarizes the four indexes created: Overall, Drought, Flood, and Wildfire PWWCIIs. Table S1 presents descriptive statistics on variables included in the PWWCIIs. While the results and analyses that follow are focused on the nationally-normed PWWCIIs, the state-normed PWWCII dataset will also be available on the CDC Tracking Network’s Interactive Data Explorer (CDC, n.d.) and available as a downloadable dataset (CDC, 2022b).

#### Census tract impact indexes:

**Fig. 2A-D.**
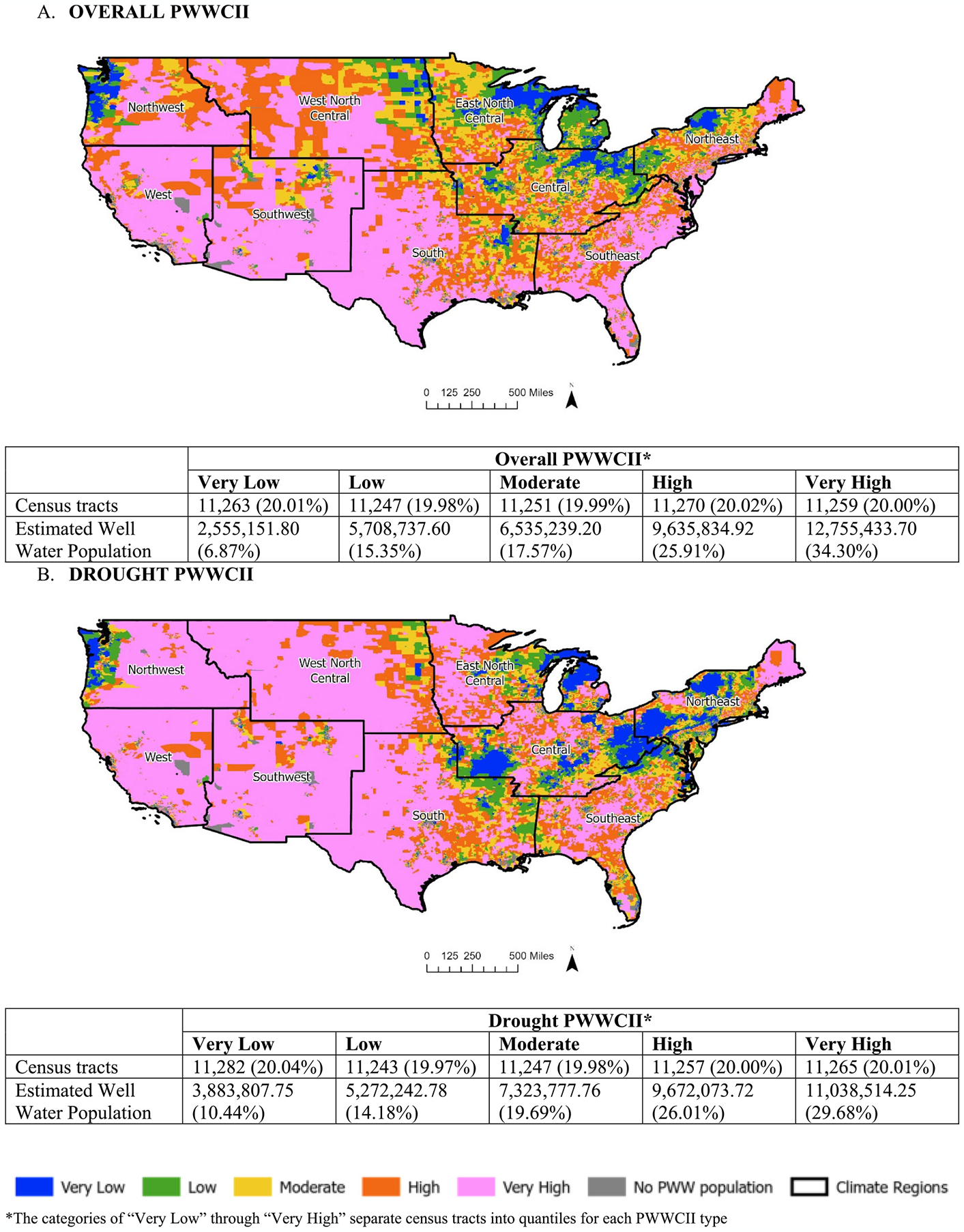

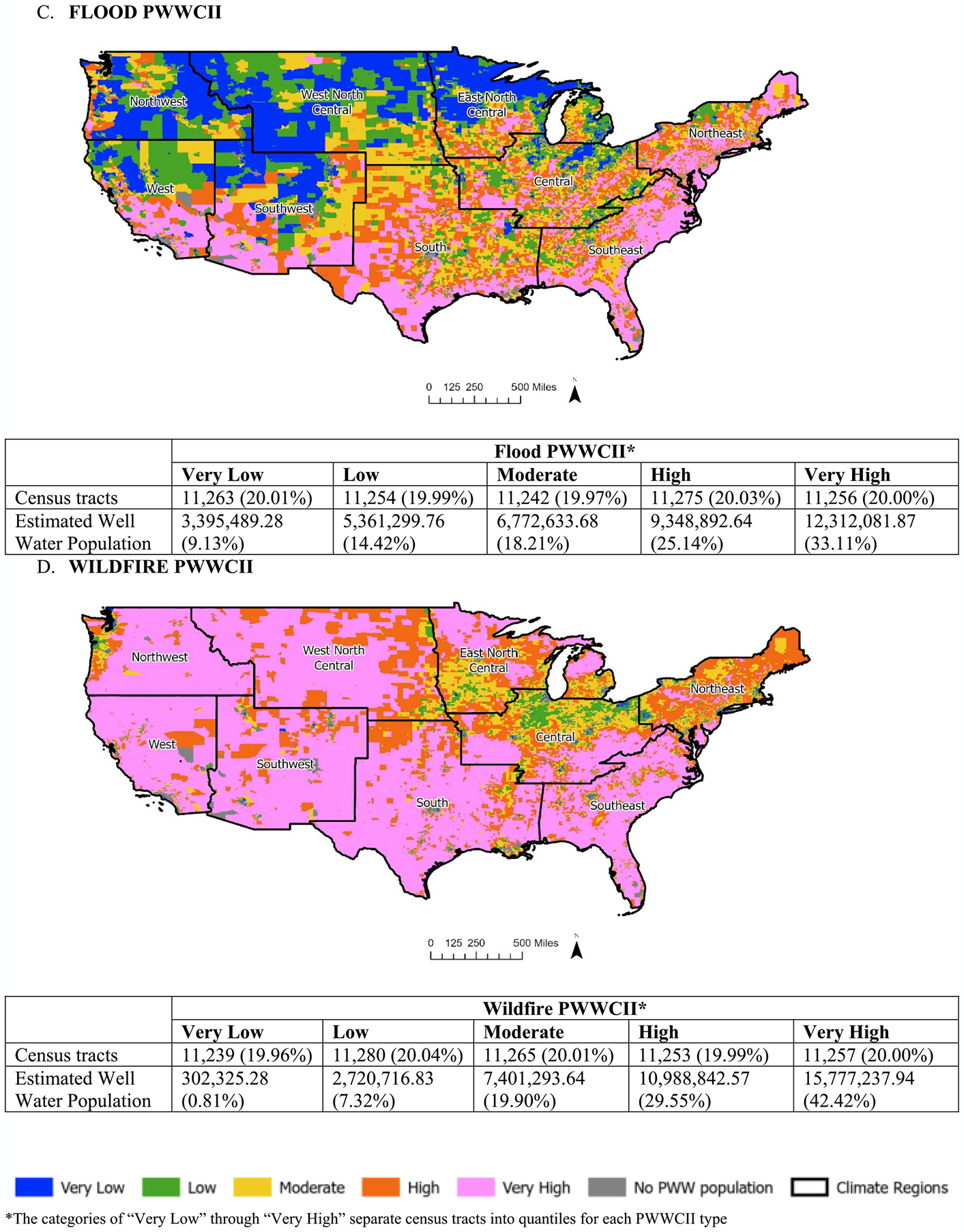
Maps of nationally-normed PWWCII census tracts across Continental United States (CONUS) by the nine climate regions.

The distribution of climate-related impact census tracts varied across CONUS by nationally-normed PWWCII type (Figs. 2A–D). While each PWWCII category (e.g., Very Low) contained approximately 20 % of PWWCII census tracts by design, estimated persons dependent upon private wells increased as impact category increased for the PWWCIIs; this was expected due to the inclusion of the census tract level variable “percent of population on private well water” in the population characteristics component of the indexes.

For the Overall PWWCII census tracts (*n* = 56,290), Very High climate-related impact census tracts were highest in the Southeast (*n* = 3616 or 6.4 %) and South (*n* = 1570 or 2.8 %) (Fig. 2A); whereas the greatest number of Very Low Overall climate-related impact census tracts were in Central (*n* = 2947 or 5.2 %) and East North Central (*n* = 2349 or 4.2 %). For the Drought PWWCII census tracts (*n* = 56,294), the West (*n* = 2540 or 4.5 %) and South (*n* = 2114 or 3.8 %) contained the largest number of Very High Drought climate-related impact census tracts (Fig. 2B); in contrast, the most Very Low Drought climate-related impact census tracts were in the Northeast (*n* = 3646 or 6.5 %) and Central (*n* = 2261 or 4.0 %). For the Flood PWWCII census tracts (*n* = 56,290), Very High climate-related impact census tracts were highest in the Southeast (*n* = 4086 or 7.3 %) and Northeast (*n* = 3137 or 5.6 %) (Fig. 2C), and the most Very Low Flood climate-related impact census tracts were in Central (*n* = 2340 or 4.2 %) and East North Central (*n* = 2289 or 4.1 %). Lastly, the largest number of Very High climate-related impact census tracts for the Wildfire PWWCII census tracts (n = 56,294) were in the Southeast (*n* = 3770 or 6.7 %) and South (*n* = 2618 or 4.7 %) (Fig. 2D); in comparison, most of the Very Low Wildfire climate-related impact census tracts were in the Northeast (*n* = 3380 or 6.0 %) and Central (*n* = 2054 or 3.6 %).

### 3.3. Index evaluation

The variable percentiles comprising each of the PWWCIIs were not highly correlated — Spearman correlations ranged from −0.38 to 0.46 (Table S2A); similarly, component percentiles for each PWWCII and PWWCII percentiles were not highly correlated — Spearman correlations ranged from −0.13 to 0.27 (Table S2B). Lastly, it was interesting to note that population characteristics percentiles were marginally more correlated with the Flood, Wildfire, and Overall PWWCII score percentiles than their respective climate-related hazard percentiles, except for Drought in which case they were equally correlated (Table S2B).

The convergent validity Spearman correlation coefficients between our PWWCIIs and CVI ranged from 0.38 to 0.55, and divergent validity Spearman correlation coefficients between our PWWCIIs and owner and renter occupied housing units with telephone service ranged from −0.10 to 0.00 (Tables S2C–D). Taken together, this initial evaluation supported the validity of the PWWCII construct.

### 3.4. 2010 estimated U.S. persons dependent on private wells and nationally normed PWWCIIs

**Fig. 3A-D.**
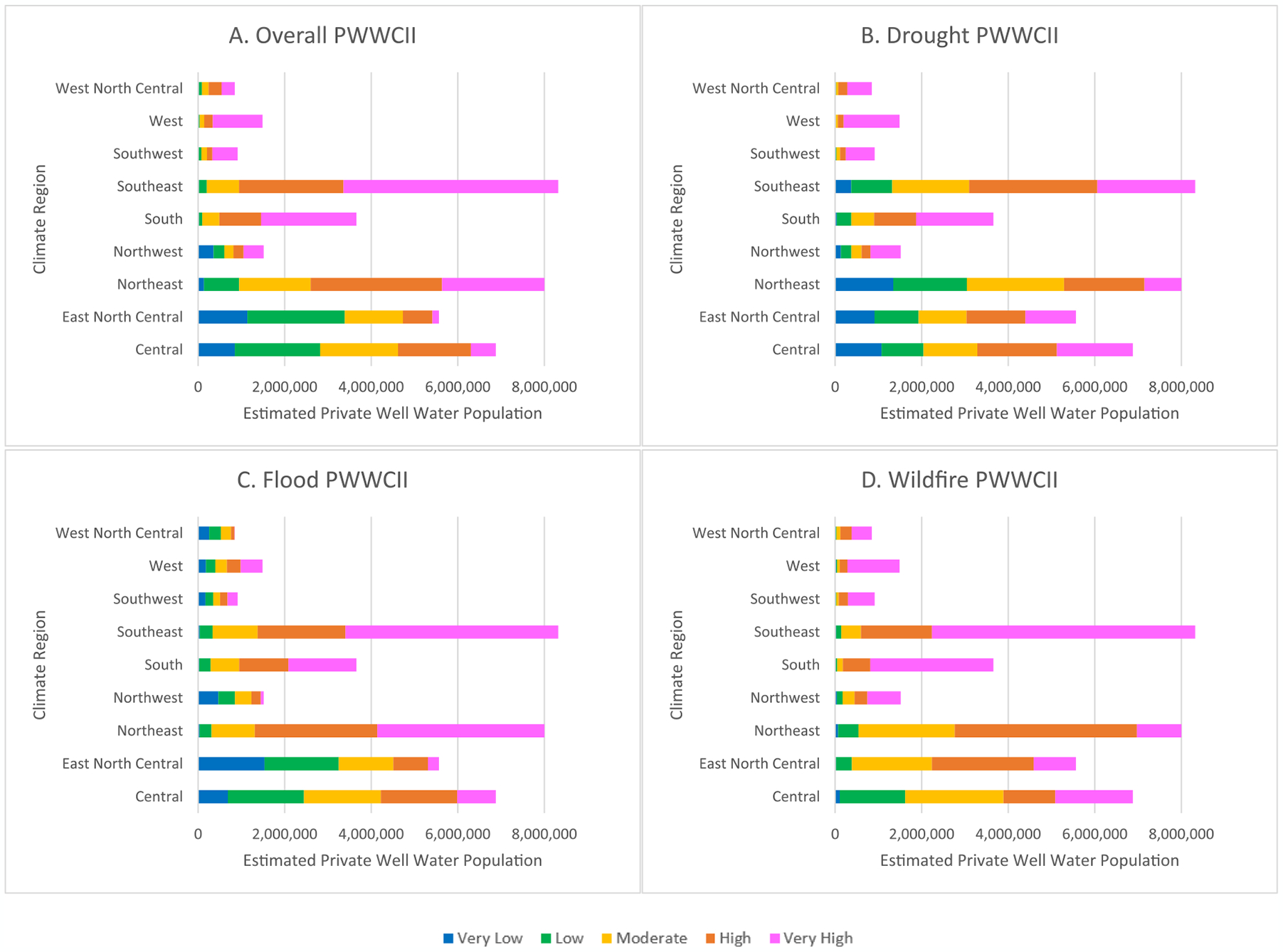
Estimated private well water population living in the nationally-normed Private Well Water Climate Impact Index (PWWCII) categories by Continental United States (CONUS) climate regions.

The distribution of the estimated private well water population living in PWWCII census tracts by climate region are visualized in Fig. 3 and presented in Table S3. For the 2010 estimated U.S. persons dependent on private wells, the univariate associations between the Overall, Flood, and Wildfire PWWCIIs and climate regions were strong (Cramer’s V = 0.32–0.34), and the univariate association between the Drought PWWCII and climate regions was moderate (Cramer’s V = 0.23) (Table 2).

Compared to the Southeast where the majority of the 2010 estimated U.S. private well water population lived, the estimated persons dependent upon private well water living in the West had an increased odds of living in higher impact census tracts for the Overall PWWCII (OR = 2.12, 95 % CI: 2.11,2.13) (Table 3 A). Whereas, for the Drought PWWCII, the estimated persons dependent on private wells living in the West (OR = 14.43, 95%CI: 14.36,14.50), Southwest (OR = 5.78, 95%CI: 5.75,5.81), West North Central (OR = 4.69, 95%CI: 4.67,4.71), South (OR = 2.09, 95%CI: 2.09,2.10), and Northwest (OR = 1.28, 95%CI: 1.27,1.28) had an increased odds of living in higher impact census tracts compared to the Southeast. For the Flood PWWCII, no climate regions in the U.S. had an increased odds of their estimated private well water population living in higher impact census tracts compared to the Southeast (Table 3 A). However, for the Wildfire PWWCII, the estimated persons dependent on private wells living in the West (OR = 1.50, 95%CI: 1.49,1.51) and South (OR = 1.31, 95%CI: 1.30,1.31) had an increased odds of living in higher impact census tracts compared to the Southeast.

### 3.5. 2010 census total U.S. population and nationally-normed PWWCIIs

**Fig. 4A-D.**
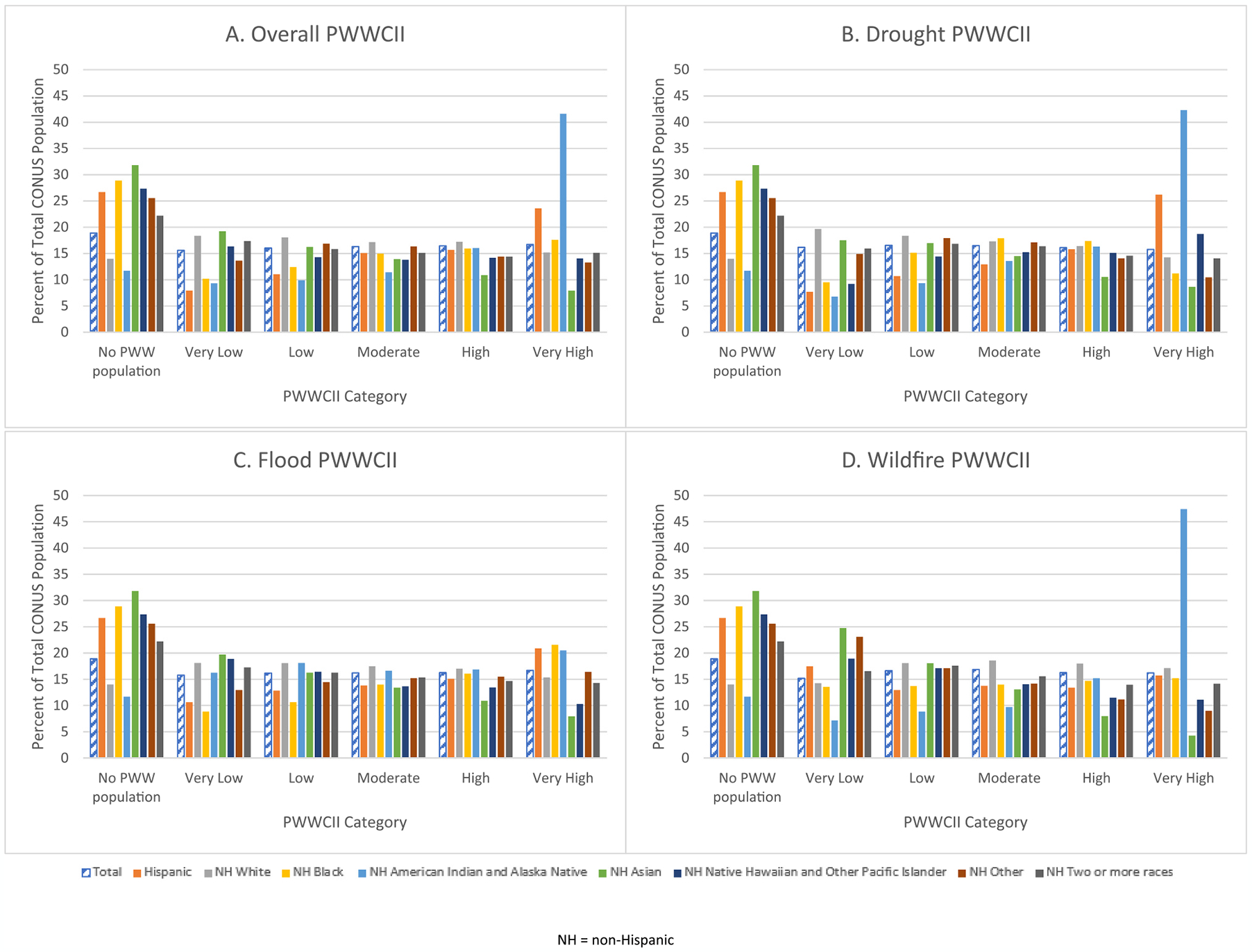
Percent of total Continental United States (CONUS) population living in the nationally-normed Private Well Water Climate Impact Index categories by race/ethnicity.

For the 2010 census total U.S. population, the univariate associations between the Overall, Drought, Flood, and Wildfire PWWCIIs and race/ethnicity were weak (Cramer’s V = 0.10–0.12); whereas, the univariate association between the four PWWCIIs and age as well as sex were negligible (Cramer’s V = 0.00–0.02) and therefore not analyzed further (Table 2). The percent of the 2010 census total U.S. population living in PWWCII census tracts and no private well water population census tracts are visualized in Fig. 4 by race/ethnicity with corresponding n’s presented in Table S4 and presented in Table S5 by sex and age.

Compared to non-Hispanic White persons, non-Hispanic American Indian and Alaska Native (AI/AN) persons (OR = 2.72, 95 % CI: 2.71,2.72) and Hispanic persons (OR = 1.06, 95 % CI: 1.06,1.06) had an increased odds of living in higher impact census tracts for the Overall PWWCII (Table 3B). Similarly non-Hispanic AI/AN persons (OR = 3.14, 95 % CI: 3.13,3.15) and Hispanic persons (OR = 1.22, 95 % CI: 1.22,1.22) had an increased odds of living in higher impact census tracts for the Drought PWWCII compared to non-Hispanic White persons. Non-Hispanic AI/AN persons (OR = 1.23, 95 % CI: 1.23,1.23) had an increased odds of living in higher impact census tracts for the Flood PWWCII (OR = 1.23, 95 % CI: 1.23,1.23) and the Wildfire PWWCII (OR = 3.12, 95 % CI: 3.11,3.13) compared to non-Hispanic White persons (Table 3B). Plots for simple ordinal regression models in sections 3.4 and 3.5 are presented in Fig. S1. With the exception of the plot for Wildfire PWWCII and Climate Region, the plots exhibit parallelism.

## 4. Discussion

Private well water is largely an under-researched area of public health in which high quality, timely, and consistent data are lacking or incomplete. We created a novel data-driven tool, the PWWCII, using novel publicly available data at a sub-county spatial resolution to characterize community-level climate-related hazards and vulnerability that impact persons dependent on private wells in communities across the continental U.S. It integrates high quality and recent data in a manner that is consistent and standardized to allow for comparison of communities across the nation and within states. The PWWCII fills a gap as it provides a baseline understanding of potential climate-related impacts to communities reliant on private well water.

With dissemination on the CDC Tracking Network’s Data Explorer (https://ephtracking.cdc.gov/DataExplorer/), the indexes can support data-informed decision making and action by the individuals, communities, as well as public officials, to protect the health of communities pre- and post-climate events who are dependent on unregulated private well water as a drinking source. Public dissemination of environmental health data on the CDC Tracking Network’s Data Explorer has also contributed to the advancement of public health research on wildfires, COVID-19, and child opportunity (Pennino et al., 2022; Gaffney et al., 2023; Noelke et al., 2020), so these data may be more accessible to researchers and public health practitioners to use the PWWCII in their work. The PWWCII is adapted from CalEnviroScreen, which has been utilized in epidemiological studies as an exposure metric to examine its association with outcomes such as pediatric asthma hospitalizations (Alcala et al., 2019) and preterm birth (Huang et al., 2018). Another area-level metric within the environmental health field, the EPA’s county-level Environmental Quality Index (EQI), has also been found to be associated with diabetes control (Jagai et al., 2021) and asthma prevalence (Gray et al., 2018). The PWWCII could similarly be explored as an exposure metric in epidemiological studies, to identify communities in which to conduct in-depth exposure studies relevant to private well water, or to identify case and control communities.

Based on the PWWCIIs we constructed, the impact to communities reliant on private well water varies substantially across the continental U.S. The Southeast contains the largest number of census tracts characterized as Very High Overall, Flood, and Wildfire impact communities, and the West contains the largest number of census tracts that are Very High Drought impact communities. In terms of estimated persons dependent on private wells, those in the West have the highest odds of living in higher impact census tracts compared to those in the Southeast for the Overall, Drought, and Wildfire PWWCIIs, which is similar to popular knowledge (Masri et al., 2023; Westerling et al., 2006).

We also examined the total U.S. population by demographic groups living in the different categories of the nationally-normed PWWCIIs because demographic data on the estimated persons dependent on private wells are lacking. Compared to non-Hispanic White persons, non-Hispanic AI/AN persons had an increased odds of living in higher impact census tracts for Overall, Drought, Flood, and Wildfire PWWCIIs. Pertaining to groups that have been historically marginalized, this may place a disproportionate burden upon communities with greater proportions of NH AI/AN residents. Additionally, compared to non-Hispanic White persons, Hispanic persons had an increased odds of living in higher impact census tracts for Overall and Drought PWWCIIs. These findings are supported by recent analyses of unregulated well water and public water system contamination with arsenic and other metals that revealed similar racial/ethnic inequalities for AI/AN persons, Hispanic persons, as well as the Southwestern population (Spaur et al., 2021; Hoover et al., 2017; Ravalli et al., 2022; Nigra et al., 2020; Martinez-Morata et al., 2022).

This work is not without limitations. First and foremost, the PWWCII is a screening tool and conversation starter among individuals, communities, as well as public officials that summarizes baseline characteristics about climate related hazards and vulnerability at the census tract-level for communities reliant on private wells using nationally available datasets. Therefore, there may be more accurate data sources at the state or local level that could be substituted in the calculations. From an ecological fallacy standpoint, it is not appropriate to use the PWWCII to characterize impact or contamination to individual private wells. PWWCII maps may be misinterpreted as individual-level and not community-level impact and risk maps; this is especially important to highlight since individual well characteristics may be more influential when it comes to contamination than the surrounding geography (Eccles et al., 2017). In summary, the PWWCII is a census tract measure and community information tool.

Data limitations exist around the private well water data: the 2010 persons dependent on private wells and geography data are modeled on information about households dependent on domestic wells from the 1990 census (Johnson et al., 2019); while the arsenic and nitrate data are available at a 1 km^2^ resolution, the authors state the machine learning modeled data may be more reliable at regional and national scales (Ransom et al., 2022; Lombard et al., 2021b); lastly, the geographic coverage of all well data is limited to CONUS and thus excludes Alaska, Hawaii, and U.S. territories. In terms of the climate data, annualized frequency data were utilized providing nationally consistent measures; however, alternative measures for low frequency, high impact climate hazards, such as hurricanes, may provide more accurate estimates (Zuzak et al., 2021). Additionally, the time periods of the data sources varied but were mapped to 2010 census boundaries. Methodologically, approximately 52,500 out of the 37.29 million people estimated to be on private well water (or 0.1 %) were excluded when converting the raster source data to area-weighted census tract populations during GIS processing.

Regarding the demographic analysis, we relied on total U.S. population data as there is no demographic data available on the persons dependent on private wells. A 2016 expert panel workshop on the public health challenge of protecting private wells determined that there are diverse populations relying on private wells for drinking water yet there is a lack of demographic data to characterize populations dependent upon private well water (Fox et al., 2016), and this remains an unmet need today. From a vulnerability standpoint, education, age, income, residence tenure, living alone, living in a rural area, and homeowner status were found to be statistically significant modifying factors associated with well testing behavior in a systematic scoping review (Colley et al., 2019), and children drinking private well water had higher blood lead levels than those on city water in North Carolina (Gibson et al., 2020). Due to the paucity of evidence on the breadth of population characteristics which may be linked to vulnerability related to private well water, CDC’s SVI was utilized due to its broad inclusion of thematic factors on socioeconomic status, household composition and disability, minority status and language, and housing type and transportation (Centers for Disease Control and Prevention/ Agency for Toxic Substances and Disease Registry/ Geospatial Research A, and Services Program, 2018). More current data on private wells Census is critical to advance public health practice and research, particularly as it relates to location, demographic, and vulnerability as well as protective factors.

We aimed to build the PWWCII because, to our knowledge, there is no published measure that currently captures the same construct, which poses a challenge to formal validation. Thus, to evaluate convergent validity, we compared the nationally-normed PWWCII but not the state-normed PWWCII to the census tract-level CVI. Additional limitations of comparison to the CVI include that the CVI does not include data on unregulated drinking water, and water quality data in general was lacking for inclusion in the CVI. There is a paucity of data available at the sub-county scale on vulnerability and climate, so our PWWCII and the CVI share source data—specifically the CDC’s SVI and FEMA’s NRI data. Nonetheless, the CVI contains 184 indicators relevant to climate change risks and baseline vulnerabilities, which is considerably more than the PWWCII contains, and the CVI adapted the Toxicological Prioritization Index (ToxPI) approach whereas the PWWCII adapted the CalEnviroScreen 4.0 approach. Despite these limitations, the nature of indexes allows for iterative improvement and expansion (e.g., CalEnviroScreen is in its fourth iteration).

## 5. Conclusions

Presently, the PWWCII is a novel, data-driven tool that can inform the protection of the public health of those communities reliant on unregulated private well drinking water from climate-related hazards in an area of public health where such tools are severely lacking. Future work could incorporate additional contaminants and natural hazards as national, sub-county data becomes available and the science of climate-related hazards and private well water contamination advances. For example, emerging evidence of potential groundwater contaminants due to wildfire could be included as more research on private well water wildfire-specific contaminants is undertaken (Pennino et al., 2022). Similarly, existing data on private well locations inclusive of Alaska and Hawaii could be incorporated if corresponding well contaminant data also become available for those states (Murray et al., 2021). While we utilized an equal weights approach to the variables included, PWWCII 2.0 could specify weights for the variables that reflect how important a given variable is as a predictor of private well water climate-related impact, and like CalEnviroScreen, formal validation of the nationally-normed and state-normed indexes could be performed as necessary data become available (Greenfield et al., 2017). The PWWCII fills a gap and generated two novel, community-level datasets, nationally-normed and state-normed, that are intended to assist environmental health practitioners with evidence-based decision making, resource allocation or attainment, and outreach efforts in the context of climate hazards. In the understudied area of unregulated private wells, the PWWCII may also be a useful metric for inclusion in exposure and epidemiological studies.

## Supplementary Material

Supplement

## Figures and Tables

**Table 1:** Source data used to build the Overall, Drought, Flood, and Wildfire Private Well Water Climate Impact Indexes (PWWCIIs).

Variable	Description (data years)	Spatial resolution	Source
**Drought-specific data**			
Drought	Annualized frequency of recorded drought occurrences in incident-days based on historical occurrences (2000–2017). See sections 9.1, 9.2, 9.3, 9.5 and 9.6 of Zuzak et al. (2021) for detailed data description.	Census tract	FEMA: Zuzak et al. (2021)
Arsenic in private well water	Boosted Regression Tree modeled probability of arsenic concentrations exceeding 10 μg/L (1970–2013) from geologic, geochemical, hydrologic, and climatic predictor variables	1 km × 1 km	USGS: Lombard et al. (2021a, 2021b)
**Flood-specific data**			
Coastal flooding	Annualized frequency of modeled coastal flooding incidents based on susceptible areas (varies). See sections 7.1, 7.2, 7.3, and 7.5 of Zuzak et al. (2021) for detailed data description.	Census tract	FEMA: Zuzak et al. (2021)
Hurricane	Annualized frequency of recorded hurricane incidents based on historical occurrences (1851–2017 for Eastern US and 1949–2017 for Western US). See sections 13.1, 13.2, 13.3, 13.5 and 13.6 of Zuzak et al. (2021) for detailed data description.	Census tract	FEMA: Zuzak et al. (2021)
Riverine flooding	Annualized frequency of riverine flooding occurrences in incident-days based on susceptible areas and historical events (1996–2019). See sections 17.1, 17.2, 17.3, 17.5 and 17.6 of Zuzak et al. (2021) for detailed data description.	Census tract	FEMA: Zuzak et al. (2021)
Nitrate in private well water	Modeled groundwater nitrate concentrations (mg/L) for domestic supply (1988–2018) from predictor variables representing well characteristics, hydrology, climate, soils, land use, nitrogen inputs, and geology	1 km × 1 km	USGS: Ransom et al. (2021)
**Wildfire-specific data**			
Wildfire	Area-weighted burn probability due to a large fire incident based on probabilistic modeling and susceptible areas. See sections 22.1, 22.2, 22.3, and 22.5 of Zuzak et al. (2021) for detailed data description.	Census tract	FEMA: Zuzak et al. (2021)
**Social vulnerability data**			
Nationally-normed and state-normed Social Vulnerability Indexes (SVIs)	Sum of 15 vulnerability variables’ percentile rankings (2018)	Census tract	CDC/ATSDR (2018)
**Population data**			
Private well water population	Block Group Method modeled 2010 population using domestic supply (‘source of water’ data from 1990 U.S. Census)	1 km × 1 km	USGS: Johnson et al. (2019)

**Table. 2:** Cramer’s V for PWWCIIs by region, race/ethnicity, sex, and age.

Variables	df	V	association
**REGION**			
Overall PWWCII categories v Climate region for 2010 estimated U.S. persons dependent on private wells	4	0.34	strong
Drought PWWCII categories v Climate region for 2010 estimated U.S. persons dependent on private wells	4	0.23	moderate
Flood PWWCII categories v Climate region for 2010 estimated U.S. persons dependent on private wells	4	0.32	strong
Wildfire PWWCII categories v Climate region for 2010 estimated U.S. persons dependent on private wells	4	0.32	strong
**RACE/ETHNICITY**			
Overall PWWCII categories v race/ethnicity for 2010 census total U.S. population	5	0.10	weak
Drought PWWCII categories v race/ethnicity for 2010 census total U.S. population	5	0.12	weak
Flood PWWCII categories v race/ethnicity for 2010 census total U.S. population	5	0.10	weak
Wildfire PWWCII categories v race/ethnicity for 2010 census total U.S. population	5	0.10	weak
**AGE**			
Overall PWWCII categories v age for 2010 census total U.S. population	4	0.02	negligible
Drought PWWCII categories v age for 2010 census total U.S. population	4	0.02	negligible
Flood PWWCII categories v age for 2010 census total U.S. population	4	0.02	negligible
Wildfire PWWCII categories v age for 2010 census total U.S. population	4	0.02	negligible
**SEX**			
Overall PWWCII categories v sex for 2010 census total U.S. population	1	0.01	negligible
Drought PWWCII categories v sex for 2010 census total U.S. population	1	0.01	negligible
Flood PWWCII categories v sex for 2010 census total U.S. population	1	0.00	negligible
Wildfire PWWCII categories v sex for 2010 census total U.S. population	1	0.01	negligible

**Table 3:** Simple ordinal regression model odd ratios and 95 % confidence intervals for (A) 2010 estimated U.S. persons dependent upon private well water by region and (B) 2010 census total U.S. population by race/ethnicity.

	Overall PWWCII			Drought PWWCII			Flood PWWCII			Wildfire PWWCII		
	OR	95%CI LL	95%CI UL	OR	95%CI LL	95%CI UL	OR	95%CI LL	95%CI UL	OR	95%CI LL	95%CI UL
A. **REGION**												
Central	0.07	0.07	0.07	0.65	0.65	0.65	0.11	0.11	0.11	0.06	0.06	0.06
East North Central	0.03	0.03	0.03	0.50	0.50	0.50	0.04	0.04	0.04	0.10	0.10	0.10
Northeast	0.29	0.29	0.29	0.35	0.35	0.35	0.72	0.72	0.73	0.11	0.11	0.11
Northwest	0.10	0.10	0.10	**1.28**	**1.27**	**1.28**	0.04	0.04	0.04	0.28	0.28	0.28
South	0.99	0.99	0.99	**2.09**	**2.09**	**2.10**	0.53	0.53	0.53	**1.31**	**1.30**	**1.31**
Southwest	0.95	0.95	0.96	**5.78**	**5.75**	**5.81**	0.12	0.12	0.12	0.76	0.76	0.76
West	**2.12**	**2.11**	**2.13**	**14.43**	**14.36**	**14.50**	0.23	0.23	0.23	**1.50**	**1.49**	**1.51**
West North Central	0.37	0.36	0.37	**4.69**	**4.67**	**4.71**	0.03	0.03	0.03	0.44	0.44	0.45
Southeast	1.00			1.00			1.00			1.00		
B. **RACE/ETHNICITY**												
Hispanic	**1.06**	**1.06**	**1.06**	**1.22**	**1.22**	**1.22**	0.91	0.91	0.91	0.61	0.61	0.61
Non-Hispanic American Indian/Alaska												
Native	**2.72**	**2.71**	**2.72**	**3.14**	**3.13**	**3.15**	**1.23**	**1.23**	**1.23**	**3.12**	**3.11**	**3.13**
Non-Hispanic Asian	0.47	0.47	0.47	0.51	0.51	0.51	0.46	0.46	0.46	0.33	0.33	0.33
Non-Hispanic Black	0.81	0.81	0.81	0.75	0.75	0.75	0.93	0.92	0.93	0.61	0.61	0.61
Non-Hispanic Other	0.73	0.73	0.73	0.70	0.70	0.70	0.81	0.81	0.81	0.47	0.47	0.48
Non-Hispanic Pacific Islander	0.67	0.67	0.68	0.91	0.91	0.92	0.58	0.58	0.58	0.50	0.50	0.50
Non-Hispanic Two or more races	0.79	0.79	0.79	0.83	0.83	0.83	0.77	0.77	0.77	0.67	0.67	0.67
Non-Hispanic White	1.00			1.00			1.00			1.00		

Bold = statistically significant at *p* = 0.05; OR = odds ratio; UL = confidence interval upper limit; LL = confidence interval lower limit.

## Data Availability

PWWCII datasets will be available on the CDC Tracking Network’s Interactive Data Explorer https://ephtracking.cdc.gov/DataExplorer/

## Equation Box 2 PWWCII Rating Equation

PWWCIIi,j=Very}High}if79<percentileofPWWCIIScorei,j≤99Hig}h}if59<percentileofPWWCIIScorei,j≤79Moderat}e}if39<percentileofPWWCIIScorei,j≤59Lo}w}if19<percentileofPWWCIIScorei,j≤39Very}Low}if0≤percentileofPWWCIIScorei,j≤19.

Where *i* denotes census tract and *j* denotes the specific index (i.e., Overall, Drought, Flood, or Wildfire.).

## HIGHLIGHTS

- PWWCIIs characterize climate-related hazards and vulnerability for private well water at the census tract-level
- PWWCIIs may be useful to practitioners’ decision making and outreach efforts in the context of climate hazards
- PWWCIIs may be useful to exposure scientists and epidemiologists in the understudied area of unregulated private wells
