# Supplementary material for: The private well water climate impact index: Characterization of community-level climate-related hazards and vulnerability in the continental United States
Source: Sci Total Environ. Author manuscript; Available in PMC 2025 Apr 11. (PMC11988540; doi:10.1016/j.scitotenv.2024.177409)

Table S1. Descriptive statistics for nationally-normed Private Well Water Climate Impact Index (PWWCII) variables

| **Variable*** | **PWWCIIs included in** | **n** | **Mean (standard deviation)** | **Median** | **Min-Max** |
| --- | --- | --- | --- | --- | --- |
| Annualized drought frequency | Drought, Overall | 56,525 | 18.02 (21.24) | 8.56 | 0-130.28 |
| Probability arsenic concentration exceeds 10 µg/L in private well water |  | 56,525 | 0.08 (0.12) | 0.04 | 0.01-0.99 |
| Annualized coastal flooding frequency | Flood, Overall | 56,525 | 0.42 (1.18) | 0 | 0-4.51 |
| Annualized hurricane frequency |  | 56,525 | 0.06 (0.08) | 0.01 | 0-0.44 |
| Annualized riverine flooding frequency |  | 56,525 | 1.96 (1.93) | 1.5 | 0-12.67 |
| Nitrate concentration (mg/L) in private well water |  | 56,521 | 1.10 (2.00) | 0.34 | 0.0002-27.66 |
| Probability of wildfire | Wildfire, Overall | 56,525 | 0.0006 (0.002) | 0.00004 | 0-0.07 |
| Social vulnerability index (national) | Drought, Flood, Wildfire, Overall | 56,294 | 7.15 (2.14) | 7.08 | 0.84-13.29 |
| Percent of population on private well water |  | 56,525 | 0.16 (0.25) | 0.03 | 0.00007-1 |

*See Table 1 for detailed description of variables

Table S2A-D. Evaluation: examination of index correlation and convergent and divergent validity

1. Spearman Correlations between variable percentiles used in each PWWCII

|  | **Drought percentile** | **Arsenic percentile** | **Riverine flooding percentile** | **Coastal flooding percentile** | **Hurricane percentile** | **Nitrate percentile** | **Wildfire percentile** | **SVI percentile** | **Percent well population percentile** |
| --- | --- | --- | --- | --- | --- | --- | --- | --- | --- |
| **Drought percentile** | 1.00 |  |  |  |  |  |  |  |  |
| **Arsenic percentile** | -0.15* | 1.00 |  |  |  |  |  |  |  |
| **Riverine flooding percentile** | 0.02* | -0.05* | 1.00 |  |  |  |  |  |  |
| **Coastal flooding percentile** | -0.03* | -0.06* | 0.11* | 1.00 |  |  |  |  |  |
| **Hurricane percentile** | -0.13* | -0.38* | 0.13* | 0.46* | 1.00 |  |  |  |  |
| **Nitrate percentile** | 0.24* | 0.15* | 0.03* | -0.16* | -0.26* | 1.00 |  |  |  |
| **Wildfire percentile** | 0.44* | -0.31* | 0.14* | 0.01* | 0.02* | 0.04* | 1.00 |  |  |
| **SVI percentile** | 0.21* | -0.03* | -0.15* | -0.03* | 0.01 | 0.05* | -0.06* | 1.00 |  |
| **Percent well population percentile** | -0.10* | -0.10* | 0.01* | -0.11* | 0.01 | -0.16* | 0.40* | -0.15* | 1.00 |

*=statistically significant at p<0.05

1. Spearman correlations between component percentiles and PWWCII percentiles

|  | **Population Characteristics percentile** | **Drought PWWCII score percentile** | **Flood PWWCII score percentile** | **Wildfire PWWCII score percentile** | **Overall PWWCII score percentile** |
| --- | --- | --- | --- | --- | --- |
| **Population Characteristics percentile** | 1.00 | 0.66** | 0.64** | 0.79** | 0.69** |
| **Drought Hazards percentile** | -0.02** | 0.66** | N/A | N/A | N/A |
| **Flood Hazards percentile** | -0.13** | N/A | 0.59** | N/A | N/A |
| **Wildfire Hazard percentile** | 0.27** | N/A | N/A | 0.76** | N/A |
| **Overall Hazards percentile** | -0.01** | N/A | N/A | N/A | 0.64** |

**=statistically significant at p<0.001
N/A=not applicable

1. Divergent Spearman Correlations between telephone service and PWWCII score percentile

|  | **Owner and renter occupied housing units with telephone service^** |
| --- | --- |
| **Drought PWWCII score percentile** | -0.10*** |
| **Flood PWWCII score percentile** | 0.00 |
| **Wildfire PWWCII score percentile** | 0.00 |
| **Overall PWWCII score percentile** | -0.02*** |

***=statistically significant at p<0.0001
^sum of 2008-2012 ACS variables QZAE003 and QZAE012

1. Convergent Spearman Correlations between Climate Vulnerability Index (CVI) Score and PWWCII score percentile

|  | **CVI Score** |
| --- | --- |
| **Drought PWWCII score percentile** | 0.38** |
| **Flood PWWCII score percentile** | 0.55** |
| **Wildfire PWWCII score percentile** | 0.43** |
| **Overall PWWCII score percentile** | 0.52** |

**=statistically significant at p<0.001

Table S3A-D. Estimated private well water population living in the nationally-normed Private Well Water Climate Impact Index (PWWCII) categories by Continental United States (CONUS) climate regions.*

*Column % = within PWWCII category; Row % = within region/CONUS

Table S4A-D. Estimated total population within Continental United States (CONUS) living in the nationally-normed Private Well Water Climate Impact Index (PWWCII) categories and no PWW population census tracts by race/ethnicity

| 1. **Overall PWWCII** | | | | | | | | | |
| --- | --- | --- | --- | --- | --- | --- | --- | --- | --- |
|  | **Total** | **Hispanic** | **NH White** | **NH Black** | **NH American Indian and Alaskan Native** | **NH Asian** | **NH Native Hawaiian and Other Pacific Islander** | **NH Other** | **NH Two or more races** |
| **No PWW population** | 57,862,497 | 13,415,105 | 27,422,761 | 10,847,015 | 249,651 | 4,424,549 | 94,562 | 152,999 | 1,255,855 |
| **Very Low** | 47,790,142 | 3,988,402 | 35,976,704 | 3,828,348 | 198,832 | 2,677,537 | 56,508 | 81,639 | 982,172 |
| **Low** | 49,112,760 | 5,538,980 | 35,398,080 | 4,660,456 | 211,104 | 2,257,312 | 49,500 | 100,948 | 896,380 |
| **Moderate** | 50,011,442 | 7,558,177 | 33,647,984 | 5,624,597 | 243,606 | 1,939,065 | 47,675 | 97,812 | 852,526 |
| **High** | 50,393,797 | 7,891,979 | 33,718,064 | 5,986,370 | 342,226 | 1,507,454 | 49,049 | 86,157 | 812,498 |
| **Very High** | 51,191,050 | 11,844,342 | 29,764,487 | 6,610,253 | 888,139 | 1,101,602 | 48,567 | 79,551 | 854,109 |

| 1. **Drought PWWCII** | | | | | | | | | |
| --- | --- | --- | --- | --- | --- | --- | --- | --- | --- |
|  | **Total** | **Hispanic** | **NH White** | **NH Black** | **NH American Indian and Alaskan Native** | **NH Asian** | **NH Native Hawaiian and Other Pacific Islander** | **NH Other** | **NH Two or more races** |
| **No PWW population** | 57,862,497 | 13,415,105 | 27,422,761 | 10,847,015 | 249,651 | 4,424,549 | 94,562 | 152,999 | 1,255,855 |
| **Very Low** | 49,533,974 | 3,851,704 | 38,504,447 | 3,575,512 | 144,632 | 2,435,326 | 31,857 | 89,248 | 901,248 |
| **Low** | 50,731,204 | 5,371,403 | 36,002,194 | 5,689,561 | 199,643 | 2,359,759 | 49,877 | 107,304 | 951,463 |
| **Moderate** | 50,506,468 | 6,496,739 | 33,898,575 | 6,722,914 | 289,470 | 2,017,661 | 52,673 | 102,652 | 925,784 |
| **High** | 49,420,749 | 7,936,335 | 32,180,721 | 6,528,511 | 347,897 | 1,466,961 | 52,158 | 84,342 | 823,824 |
| **Very High** | 48,315,603 | 13,166,120 | 27,927,478 | 4,193,591 | 902,272 | 1,203,347 | 64,734 | 62,574 | 795,487 |

| 1. **Flooding PWWCII** | | | | | | | | | |
| --- | --- | --- | --- | --- | --- | --- | --- | --- | --- |
|  | **Total** | **Hispanic** | **NH White** | **NH Black** | **NH American Indian and Alaskan Native** | **NH Asian** | **NH Native Hawaiian and Other Pacific Islander** | **NH Other** | **NH Two or more races** |
| **No PWW population** | 57,862,497 | 13,415,105 | 27,422,761 | 10,847,015 | 249,651 | 4,424,549 | 94,562 | 152,999 | 1,255,855 |
| **Very Low** | 48,339,913 | 5,351,162 | 35,469,932 | 3,313,651 | 346,212 | 2,740,566 | 65,181 | 77,519 | 975,690 |
| **Low** | 49,577,713 | 6,439,918 | 35,427,096 | 4,003,009 | 386,315 | 2,259,232 | 56,899 | 86,539 | 918,705 |
| **Moderate** | 49,686,995 | 6,948,263 | 34,261,597 | 5,255,004 | 354,723 | 1,863,801 | 47,200 | 91,081 | 865,326 |
| **High** | 49,798,198 | 7,582,658 | 33,337,506 | 6,035,163 | 359,434 | 1,514,530 | 46,375 | 92,712 | 829,820 |
| **Very High** | 51,096,372 | 10,499,879 | 30,009,188 | 8,103,197 | 437,223 | 1,104,841 | 35,644 | 98,256 | 808,144 |

| 1. **Wildfire PWWCII** | | | | | | | | | |
| --- | --- | --- | --- | --- | --- | --- | --- | --- | --- |
|  | **Total** | **Hispanic** | **NH White** | **NH Black** | **NH American Indian and Alaskan Native** | **NH Asian** | **NH Native Hawaiian and Other Pacific Islander** | **NH Other** | **NH Two or more races** |
| **No PWW population** | 57,862,497 | 13,415,105 | 27,422,761 | 10,847,015 | 249,651 | 4,424,549 | 94,562 | 152,999 | 1,255,855 |
| **Very Low** | 48,339,913 | 5,351,162 | 35,469,932 | 3,313,651 | 346,212 | 2,740,566 | 65,181 | 77,519 | 975,690 |
| **Low** | 49,577,713 | 6,439,918 | 35,427,096 | 4,003,009 | 386,315 | 2,259,232 | 56,899 | 86,539 | 918,705 |
| **Moderate** | 49,686,995 | 6,948,263 | 34,261,597 | 5,255,004 | 354,723 | 1,863,801 | 47,200 | 91,081 | 865,326 |
| **High** | 49,798,198 | 7,582,658 | 33,337,506 | 6,035,163 | 359,434 | 1,514,530 | 46,375 | 92,712 | 829,820 |
| **Very High** | 51,096,372 | 10,499,879 | 30,009,188 | 8,103,197 | 437,223 | 1,104,841 | 35,644 | 98,256 | 808,144 |

Table S5A-D. Percent of total Continental United States (CONUS) population living in the nationally-normed Private Well Water Climate Impact Index (PWWCII) categories and no PWW population census tracts by sex and age.

| 1. **Overall PWWCII** | | | | | | | |
| --- | --- | --- | --- | --- | --- | --- | --- |
|  | **Total** | **Male** | **Female** | **Under Age 5** | **Age 5 to 24** | **Age 25 to 64** | **Age 65 & older** |
| **No PWW population** | 15.60 | 18.77 | 19.00 | 19.22 | 19.22 | 19.17 | 16.88 |
| **Very Low** | 16.03 | 15.52 | 15.68 | 14.80 | 15.27 | 15.95 | 15.27 |
| **Low** | 16.32 | 16.00 | 16.06 | 15.37 | 15.73 | 16.13 | 16.61 |
| **Moderate** | 16.45 | 16.32 | 16.33 | 16.24 | 16.35 | 16.23 | 16.70 |
| **High** | 16.71 | 16.49 | 16.41 | 16.46 | 16.38 | 16.26 | 17.36 |
| **Very High** | 18.89 | 16.91 | 16.52 | 17.91 | 17.06 | 16.27 | 17.18 |

| **B. Drought PWWCII** | | | | | | | |
| --- | --- | --- | --- | --- | --- | --- | --- |
|  | **Total** | **Male** | **Female** | **Under Age 5** | **Age 5 to 24** | **Age 25 to 64** | **Age 65 & older** |
| **No PWW population** | 18.89 | 18.77 | 19.00 | 19.22 | 19.22 | 19.17 | 19.17 |
| **Very Low** | 16.17 | 16.07 | 16.26 | 14.76 | 15.42 | 16.52 | 16.52 |
| **Low** | 16.56 | 16.48 | 16.64 | 15.81 | 16.21 | 16.73 | 16.73 |
| **Moderate** | 16.49 | 16.45 | 16.52 | 16.34 | 16.36 | 16.49 | 16.49 |
| **High** | 16.13 | 16.20 | 16.07 | 16.42 | 16.33 | 15.91 | 15.91 |
| **Very High** | 15.77 | 16.04 | 15.51 | 17.45 | 16.46 | 15.18 | 15.18 |

| **C. Flood PWWCII** | | | | | | | |
| --- | --- | --- | --- | --- | --- | --- | --- |
|  | **Total** | **Male** | **Female** | **Under Age 5** | **Age 5 to 24** | **Age 25 to 64** | **Age 65 & older** |
| **No PWW population** | 18.89 | 18.77 | 19.00 | 19.22 | 19.22 | 19.17 | 16.88 |
| **Very Low** | 15.78 | 15.78 | 15.78 | 15.36 | 15.54 | 16.10 | 15.18 |
| **Low** | 16.18 | 16.17 | 16.19 | 15.70 | 16.05 | 16.19 | 16.66 |
| **Moderate** | 16.22 | 16.24 | 16.20 | 16.01 | 16.18 | 16.11 | 16.83 |
| **High** | 16.25 | 16.28 | 16.24 | 16.19 | 16.13 | 16.10 | 17.18 |
| **Very High** | 16.68 | 16.77 | 16.59 | 17.52 | 16.87 | 16.33 | 17.26 |

| **D. Wildfire PWWCII** | | | | | | | |
| --- | --- | --- | --- | --- | --- | --- | --- |
|  | **Total** | **Male** | **Female** | **Under Age 5** | **Age 5 to 24** | **Age 25 to 64** | **Age 65 & older** |
| **No PWW population** | 18.89 | 18.77 | 19.00 | 19.22 | 19.22 | 19.17 | 16.88 |
| **Very Low** | 15.17 | 15.08 | 15.27 | 14.96 | 15.06 | 15.51 | 14.17 |
| **Low** | 16.62 | 16.50 | 16.75 | 16.30 | 16.65 | 16.65 | 16.62 |
| **Moderate** | 16.84 | 16.81 | 16.86 | 16.76 | 16.93 | 16.73 | 17.12 |
| **High** | 16.26 | 16.32 | 16.20 | 16.26 | 16.18 | 16.08 | 17.21 |
| **Very High** | 16.22 | 16.52 | 15.92 | 16.50 | 15.97 | 15.87 | 18.00 |

Figure S1. Simple Ordinal Regression Models: Empirical Cumulative Logits for PWWCII types by Climate Region and Race/Ethnicity


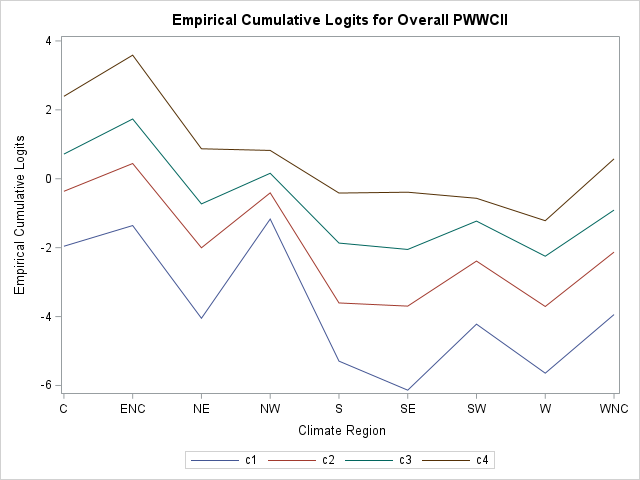


Figure Abbreviations: Central (C), East North Central (ENC), Northeast (NE), Northwest (NW), South (S), Southeast (SE), Southwest (SW), West (W), West North Central (WNC)


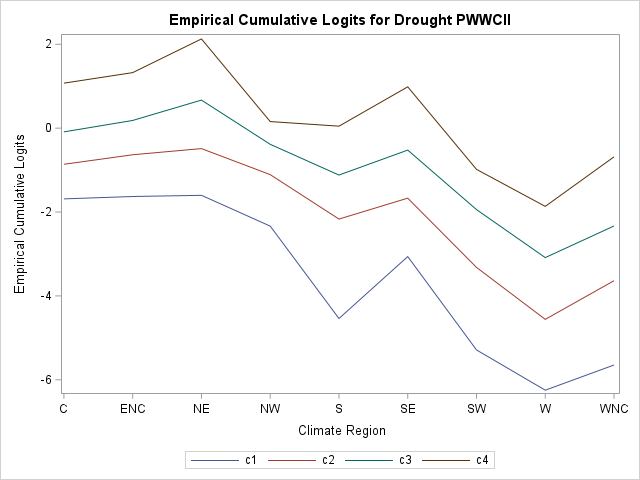


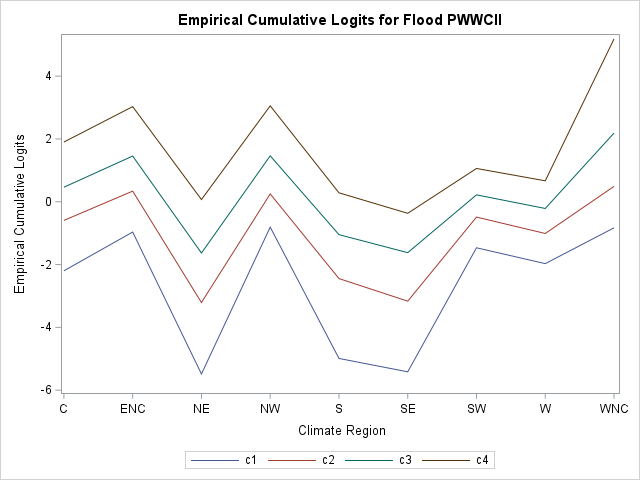


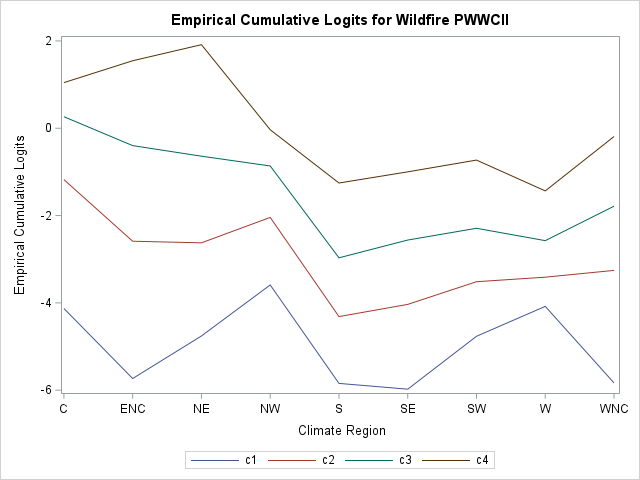


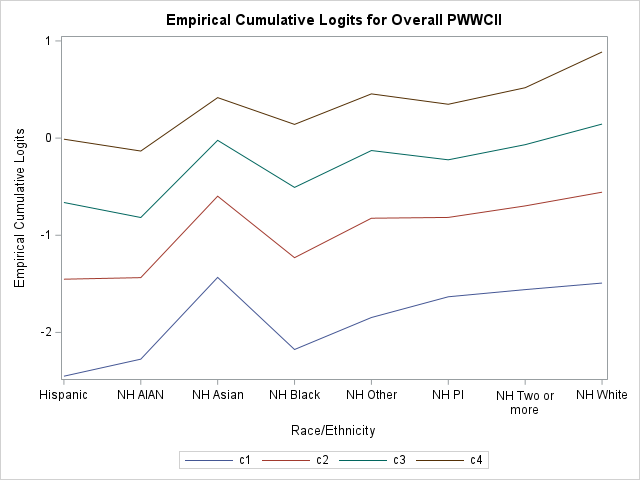


Figure Abbreviations: Non-Hispanic (NH), American Indian and Alaska Native (AIAN), PI (Pacific Islander)


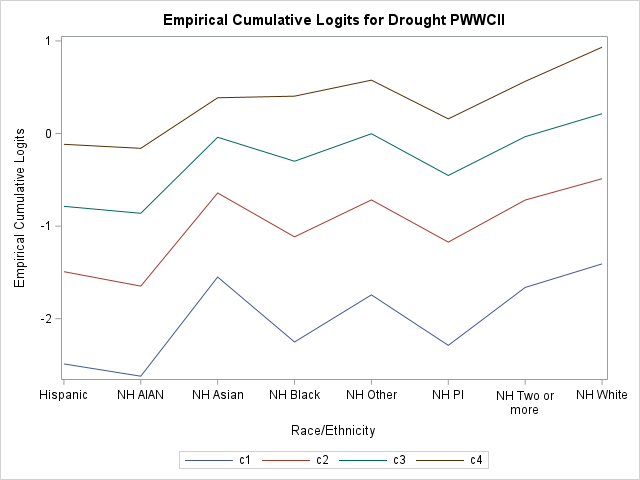


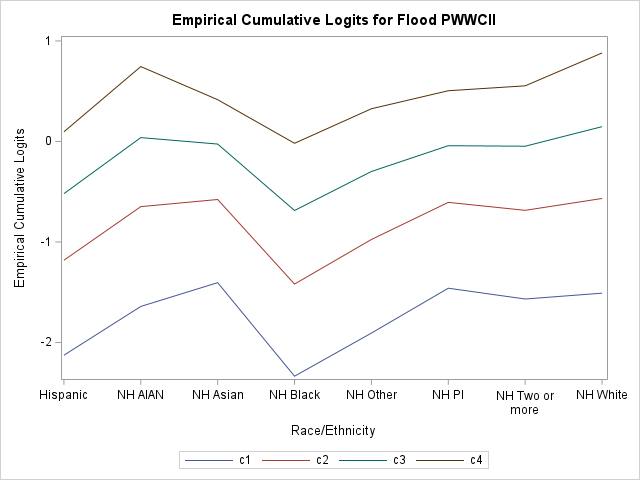


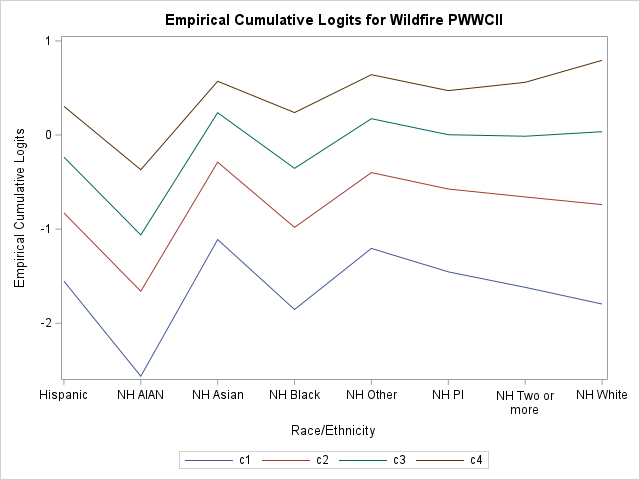

Supplement: Supplement [file NIHMS2070861-supplement-Supplement.docx]
